# Transcription Analysis of the Stress and Immune Response Genes to Temperature Stress in *Ostrinia furnacalis*

**DOI:** 10.3389/fphys.2019.01289

**Published:** 2019-10-15

**Authors:** Kangkang Chen, Tai Tang, Qisheng Song, Zhenying Wang, Kanglai He, Xu Liu, Jiahui Song, Libao Wang, Yizhong Yang, Congjing Feng

**Affiliations:** ^1^Department of Plant Protection, College of Horticulture and Plant Protection, Yangzhou University, Yangzhou, China; ^2^Division of Plant Sciences, University of Missouri, Columbia, MO, United States; ^3^State Key Laboratory for Biology of Plant Diseases and Insect Pests, Institute of Plant Protection, Chinese Academy of Agricultural Sciences, Beijing, China

**Keywords:** *Ostrinia furnacalis*, temperature stress, immunity, heat shock protein, Ferritin

## Abstract

*Ostrinia furnacalis* is one of the most important pests on maize. *O. furnacalis* larvae are frequently exposed to the temperature challenges such as high temperature in summer and cold temperature in winter in the natural environment. High and low temperature stress, like any abiotic stress, impairs the physiology and development of insects. Up to now, there is limited information about gene regulation and signaling pathways related to the high and cold stress response in *O. furnacalis.* High-throughput sequencing of transcriptome provides a new approach for detecting stress and immune response genes under high and low temperature stresses in *O. furnacalis*. In the present study, *O. furnacalis* larvae were treated with the temperature at 8 and 40°C, and the responses of *O. furnacalis* larvae to the temperature stress were investigated through RNA-sequencing and further confirmation. The results showed that immune responses were up-regulated in larvae by the cold stress at 8°C while some stress response genes, such as *HSP* family, *GST*-2, *Bax inhibitor* and *P450*, were significantly increased at 40°C. Furthermore, quantitative real time polymerase chain reaction were performed to quantify the expression levels of immune related genes, such as *PGRP-LB*, antimicrobial peptides, lysozyme, serine protease and stress response genes such as small *HSP*s and *HSP*90, and the expression levels of these genes were similar to the RNA-seq results. In addition, the iron storage protein Ferritin was found to be involved in the response to temperature stress, and the changes of total iron concentration in the hemolymph were, in general, consistent with the expression levels of *Ferritin*. Taken together, our results suggested that the stress response genes were involved in the defense against the heat stress at 40°C, and the immune responses triggered by cold stress might provide protection for larvae from cold stress at 8°C. More interestingly, our results showed that during the responses to temperature stress, the total iron concentration in hemolymph regulated by Ferritin increased, which might help *O. furnacalis* in surviving the low and high temperature stress.

## Introduction

Insects belong to ectotherms, which have weak thermoregulation, so that extreme temperatures could cause great changes of development, and even survival rate of insects (Dunkov et al., 2002). Most insects live in the wild and evolve as the master of survival during the combat with harsh environment, natural enemy and invading microorganisms (Sinclair et al., 2013). To defend against the variable temperature and pathogenic challenges such as bacterial and fungal infections, insects form fast response mechanisms (Thomas and Blanford, 2003; Sinclair et al., 2013; Wojda, 2017). Cold or heat stress often results in changes of energy consumption, metabolic rate, development and innate immune response (Williams et al., 2012; Sinclair et al., 2013). In the cold winter, many insects enter diapause to overwinter (Sinclair et al., 2013). On the other side, heat stress genes and antioxidant genes, such as HSPs, superoxide dismutase and catalase, are up-regulated (Kang et al., 2017) when the insects are under high temperature stress, especially in hot summer (Lopez-Martinez and Denlinger, 2008).

Immune responses of insects are correlated with ambient temperature. Equipped with innate immunity, insects possess cellular immunity and humoral immunity to fight against the invading pathogens such as bacteria, fungi, viruses and parasites for survival (Hoffmann, 1995; Hoffmann and Reichhart, 2002; Pal and Wu, 2009; Hoffmann and Akira, 2013; Li et al., 2016; Chen and Lu, 2018; Feng et al., 2018). Beside pathogenic infections, extreme temperatures are also the triggers of innate immune response in insects, and there is a positive relationship between temperature and immune responses (Thomas and Blanford, 2003; Wojda, 2017). In *Tenebrio molitor* larvae, the immune response of lipopolysaccharide (LPS) treated group at 30°C was stronger than that treated at lower temperatures such as 10 or 20°C, while the metabolic rate of LPS treated group at 30°C remained unchanged compared to control, but the metabolic rate of LPS treated at 10 or 20°C was significantly higher than that of controls (Catalan et al., 2012). Therefore, the environmental temperature is a key abiotic factor related to the efficiency for biocontrol of pests by fungi and bacteria. Interestingly, the regulation of body temperature through behaviors in some insects contributed to their survival after infected with entomopathogen (Arthurs and Thomas, 2001), and upregulation of antioxidant genes such as catalase and superoxide dismutase-2 helped *Culex pipiens* overwinter (Sim and Denlinger, 2011). In insects, HSPs were expressed constitutively and up-regulated by environmental stresses such as heat and cold (King and MacRae, 2015). Small heat shock proteins (sHsps) were involved in cell defense by protecting the substrate proteins (Basha et al., 2012; Arrigo, 2013), and other three families of HSPs, including HSP60, HSP70 and HSP90 interrelated with other proteins to regulate the rate of protein synthesis, cell signaling, transcription, and metabolism in insects (Robich et al., 2007). In *Sarcophaga crassipalpis*, in response to cold hardening (Robich et al., 2007), HSP26 was up-regulated in brain (Li and Denlinger, 2008). In *Liriomyza sativa* and *Liriomyza huidobrensis*, except for HSP60, all of HSP40, HSP20, HSP70, and HSP90 were induced by both heat and cold stresses (Huang and Kang, 2007; Huang et al., 2009). In *Drosophila*, as response proteins to 39°C heat stress, HSP68 contributed to variation under high temperature stress (McColl et al., 1996). Upon heat stress, *Lucilia cuprina* enhanced the expression of HSP60 with function as thermo-tolerance factor to suit the high temperature environment (Kumar Singh et al., 2015). Bax inhibitor was a conserved protein to suppress proapoptotic protein Bax, and eventually to inhibit cell death in plants and mammals (Huckelhoven, 2004; Ishikawa et al., 2011). Recently, some investigations have shown that Bax inhibitor as a cytoprotective protein was involved in the responses to heat stress in plant and fungus (Chen et al., 2015; Lu et al., 2018). Up-regulated Bax inhibitor induced by heat stress was involved in the upregulation of heat-responsive genes such as s*HSP*, *HSP70B*, and *HSP90.1* to enhance thermotolerance in wheat, *Triticum aestivum* (Lu et al., 2018). These data indicate that Bax inhibitor is also an important heat-responsive protein under heat stress to contribute to thermotolerance.

Iron starvation regulated by ferritin and transferrin of insects was an efficient strategy to kill invading pathogens (Geiser and Winzerling, 2012; Otho et al., 2016). As an essential nutrient, iron acquisition is required for both insects and invading pathogens. Through the regulation of iron distribution by ferritin and transferrin in hosts, iron is involved in electron transfer, DNA synthesis, development, cell cycle, energy metabolisms (Theil, 1987). In insects, iron is strictly limited, especially when insects are infected by bacteria. Silkworm ferritin was up-regulated by bacterial infection to decrease the iron concentration of hemolymph, leading to the elimination of the invading bacteria due to iron starvation (Otho et al., 2016). Therefore, to acquire iron from insects, the invading bacteria must break the iron barriers constructed by insects (Cassat and Skaar, 2013). Ferritin were induced by heat to enhance tolerance to heat stress in *T. aestivum* (Zang et al., 2017), suggesting there might be a cross-talk between environmental temperature and iron regulation by ferritin and transferrin in hosts.

*Ostrinia furnacalis* is an important pest on maize all over Asia (Guo et al., 2017), damages all the developmental stages of maize in hot summer, and causes great yield loss every year. *O. furnacalis* larvae can overwinter under low temperature (Lopez-Martinez and Denlinger, 2008; Wang et al., 2014; Shang et al., 2015). However, the mechanism of *O. furnacalis* in response to temperature stress is still not clearly deciphered. In this study, we focused on the responses of *O. furnacalis* larvae to certain treatment under the extreme temperature stresses. Based on the analysis of RNA-seq data and further investigation, we found immune responses were triggered by the cold environment, and some stress response genes such as *HSP*s and P450 related proteins could protect *O. furnacalis* larvae from high temperature challenges.

## Materials and Methods

### Experimental Insect and Temperature Stress Treatment

*Ostrinia furnacalis* larvae were reared over 10 years under insectary conditions at 25 ± 1°C, RH > 80% and with a photoperiod of 16 h light and 8 h darkness. The larval instars were determined by the head width as described previously (Wu et al., 2018). To understand the responses of *O. furnacalis* larvae to temperature stress, four groups of day 1, 5th instar larvae were reared at 8 and 40°C for 1 and 2 h, respectively, and the control group were chosen from the larvae under insectary conditions at 25°C (three biological replicates in each group, three larvae in each group). The whole larvae in each group were frozen in liquid nitrogen immediately for RNA isolation and other subsequent analyses.

### Library Construction and RNA-Sequencing

To minimize the variation between samples in each group, the harvested samples in each group were pooled together, and the total RNA samples were isolated according to the protocol of Trizol (Invitrogen, Carlsbad, United States). The genomic DNA was removed using DNase I (Invitrogen, Carlsbad, United States). The concentration and the integrity of the RNA samples were measured by Nanodrop ND-2000 spectrophotometer (NanoDrop, United States) and Agilent 2100 BioAnalyzer (Agilent, Palo Alto, United States), respectively. Total RNA (10 μg) in each group was used for constructing RNA-seq libraries following the manufacturer’s instructions of RNA-Seq Library Preparation Kit for Whole Transcriptome Discovery Kit (Gnomegen, San Diego, United States). RNA-seq libraries were sequenced on Illumina HiSeq^TM^ 2000 platform (Illumina, San Diego, United States).

### *De novo* Assembly and Annotation of Transcripts

After removal of low quality tags, such as tag sequences, adaptor sequences and contaminated reads, the clean reads were assembled to contigs using Trinity (Grabherr et al., 2011). The contigs in each library were pooled together to generate the unigenes (Li and Godzik, 2006). The databases from NR, NT, KO, SwissProt, PFAM, GO and KOG were used for annotation of all the assembled unigenes through BLASTX (*E*-value ≤ 1e^–5^) (Xiong et al., 2015).

### Differential Expression Analysis

Clean reads were mapped to the assembled unigenes using Bowtie software with the default parameters (Langmead et al., 2009). RSEM, the utility package of the Trinity software was used to calculate the gene expression value FPKM (fragments per kilobase per million mapped reads), and the FPKM in each group was submitted to DEGseq package for calculation of differentially expressed transcripts in R environment (*p*-value < 0.001, fold change > 2) (Wang et al., 2010).

### Quantitative Real-Time Polymerase Chain Reaction (qPCR)

To examine expression levels of various genes in day 1, 5th instar larvae, qPCR was performed using the specific primers (Supplementary Table S1). All primers in this study were designed using Primer-BLAST^1^. The whole bodies of larvae were collected and homogenized in Trizol solution (Invitrogen, Shanghai, China) for total RNA isolation. The total RNA samples were treated with RNase-free DNase I (Promega) to remove genomic DNA. First-strand cDNA was synthesized from 1.0 μg of total RNA using PrimeScript^TM^ RT Reagent Kit (TaKaRa) according to the manufacturer’s protocol. The gene encoding ribosomal protein L8 (*rpL*8) was used as an internal control for *O. furnacalis* gene transcript level comparison in all the experiments as previously described (Feng et al., 2011). qPCR was carried out on a Bio-Rad CFX96 Real Time Detection System (Bio-rad, CA, United States) in 20 μL reaction containing 1 μL of cDNA from each tissue, 10 μL of SSOfast SYBR Green Mix, 1.0 μL of each primer (20 pmol/μL) and 7 μL ddH_2_O. Thermal cycling conditions were: initial denaturation at 95°C for 3 min, followed by 40 cycles of denaturation at 95°C for 15 s, annealing at 60°C for 15 s and extension at 72°C for 15 s, melting curve performed from 60 to 95°C. qPCR data were submitted to GraphPad for figure plotting and statistical analysis (Student’s *t*-test, asterisks indicate significant differences: ^∗^*p* < 0.05; ^∗∗^*p* < 0.01; ^∗∗∗^*p* < 0.001) (Chen et al., 2014).

### Total Iron Concentration Assay in Larval Plasma

To analyze the changes of total iron concentration in response to temperature stress, the *O. furnacalis* larvae were treated at 40 and 8°C for 1 or 2 h and then bled for hemolymph collection. To remove the hemocytes, the collected hemolymph was centrifuged at 500 *g* for 10 min at 4°C. The plasma was used for total iron assay according to the protocol QuantiChrom^TM^ Iron Assay Kit (BioAssay Systems). In brief, after the samples were incubated with working reagent prepared in the kit for 40 min at 25°C, Fe^3+^ in the samples was reduced to Fe^2+^ with the reductant in the kit, and the resulting Fe^2+^ reacted with chromogen provided in the kit to form a colored complex (Otho et al., 2016). Finally, the mixture was transferred to cuvettes for optical density detection at 590 nm, and the optical density of the colored complex could be converted to total iron concentration of the plasma based on the standard curve of iron.

## Results

### Characterization of *O. furnacalis* Transcriptome by Illumina RNA Sequencing

In this study, the whole larvae treated with low and high temperatures were collected for RNA extraction and further cDNA library construction. Five cDNA libraries were sequenced on Illumina HiSeq^TM^ 2000 platform and 55.6 (OfL_25_0), 54.3 (OfL_40_1), 55.4 (OfL_40_2), 54.9 (OfL_8_1) and 55.0 (OfL_8_2) million high-quality reads were obtained, respectively (Table 1). All the clean reads were pooled together for *de novo* assembly using the Trinity software and finally 81,467 unigenes with mean lengths of 786 nt, total bases of 64,048,359 nt, and N50 length of 1636 nt (Supplementary Tables S2, S3) were produced.

**TABLE 1:** Quality control of DGE libraries.

**Sample**	**Raw Reads**	**Clean Reads**	**Clean bases**	**Error (%)**	**Q20 (%)**	**Q30 (%)**	**GC (%)**
OfL_25_0	55788684	55613718	6.96 G	0.03	97.21	93.62	44.79
OfL_40_1	54440082	54305948	6.78 G	0.03	97.35	93.79	45.39
OfL_40_2	55597160	55421398	6.92 G	0.03	97.28	93.73	45.22
OfL_8_1	55143666	54949084	6.86 G	0.03	97.31	93.74	45.26
OfL_8_2	55180336	55043008	6.88 G	0.03	97.47	94.03	45.1

*The “OfL_25_0” refers to the normal temperature group under 25°C; the “OfL_40_1” refers to the high temperature treated group 1 h posttreatment under 40°C; the “OfL_40_2” refers to the high temperature treated group 2 h posttreatment under 40°C; the “OfL_8_1” refers to the low temperature treated group 1 h posttreatment under 8°C; the “OfL_8_2” refers to the low temperature treated group 2 h posttreatment under 8°C. Q20/Q30 stands for the percent of bases with quality score [–10 × lg (error rate)] more than 20 and 30 (indicating error rates of 1% and 1‰, respectively).*

### Annotation of Unigenes and Differential Expression Analysis

To further understand the responses of *O. furnacalis* under temperature stress condition, the assembled unigenes were submitted to BLASTX for annotation. All the 81,467 unigenes were annotated in NR, NT, KO, SwissProt, PFAM, GO or KOG databases (Supplementary Tables S4, S5). After pairwise comparisons between temperature stress treated group and control group, 1,950 differentially expressed transcripts (DET, *p*-value < 0.05 and fold change > 2) were identified (Supplementary Table S6).

**FIGURE 1:**
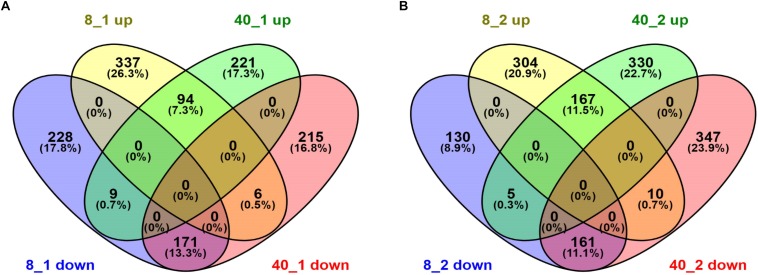
Venn diagram of differentially expressed transcripts in *O. furnacalis* larvae after temperature stress. Venn diagram of differentially expressed transcripts in *O. furnacalis* larvae at 1 h **(A)** and 2 h **(B)** posttreatment with low and high temperatures. Numbers of up- and down-regulated transcripts were indicated. All the FPKM values of extreme temperature treated groups were normalized by the FPKM values in 25°C treated groups. 8_1 up or down: Up or down-regulated transcripts at 1 h posttreated under 8°C; 8_2 up or down: Up or down-regulated transcripts at 2 h posttreated under 8°C; 40_1 up or down: Up or down-regulated transcripts at 1 h posttreated under 40°C; 40_2 up or down: Up or down-regulated transcripts at 2 h posttreated under 40°C.

To uncover the transcriptional patterns of these differentially expressed transcripts in *O. furnacalis* in response to temperature stress conditions, these differentially expressed transcripts were submitted to the website http://bioinfogp.cnb.csic.es/tools/venny/index.html for Venn analysis. We found both the up-regulated transcripts and down-regulated transcripts in 8°C group were more than that in 40°C group after 1 h treatment (Figure 1A), but were less after 2 h treatment (Figure 1B), suggesting that the response of *O. furnacalis* larvae to low temperature treatment was stronger than that to high temperature treatment at 1 h posttreatment, while the opposite is true at 2 h posttreatment.

**FIGURE 2:**
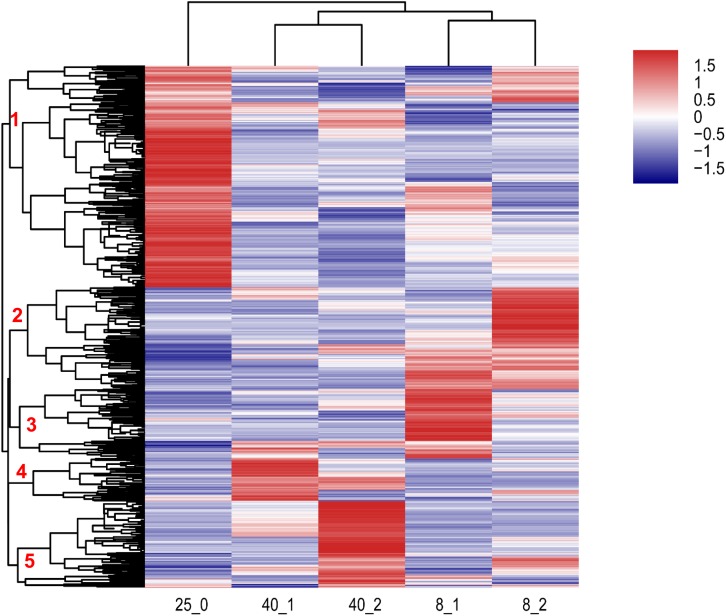
Hierarchical clustering analysis of differentially expressed transcripts in *O. furnacalis* larvae after treated with low and high temperatures. Hierarchical clustering analysis of all the differentially expressed transcripts (fold change > 2, *p*-value < 0.001) in *O. furnacalis* larvae. The larvae were treated with temperature stress under 8 and 40°C, respectively, and the whole bodies of the treated larvae were collected 1 and 2 h posttreatment for RNA extraction and library construction. 8_1 and 8_2: The differentially expressed transcripts in the low temperature (8°C) treated group 1 and 2 h posttreatment; 40_1 and 40_2: The differentially expressed transcripts in the high temperature (40°C) treated group 1 and 2 h posttreatment; 25_0: The differentially expressed transcripts in the normal temperature group under 25°C. The detail information of various genes in the heatmap was provided in Additional file 5 Supplementary Table S7.

Two-dimensional hierarchical clustering was performed for analysis of 1,950 differentially expressed transcripts using “pheatmap” package in R language. These transcripts were divided into 5 clusters in the row level and 3 clusters based on the temperature of treatments (Figure 2). The transcripts in Cluster 1 were up-regulated in 25°C group, the transcripts in Cluster 2 and 3 were up-regulated at 8°C after 1 and 2 h treatment, respectively. The transcripts in Cluster 4 and 5 were up-regulated at 40°C after 2 and 1 h treatment, respectively. These data suggested that the transcripts in Cluster 1 were required for normal development at 25°C, the transcripts in Cluster 2, Cluster 3, Cluster 4 and Cluster 5 were extreme temperature-responsive genes (Figure 2), and the responding mechanisms to different extreme temperatures in *O. furnacalis* varied. The detail information of various genes in the heatmap was provided in Supplementary Table S7.

**FIGURE 3:**
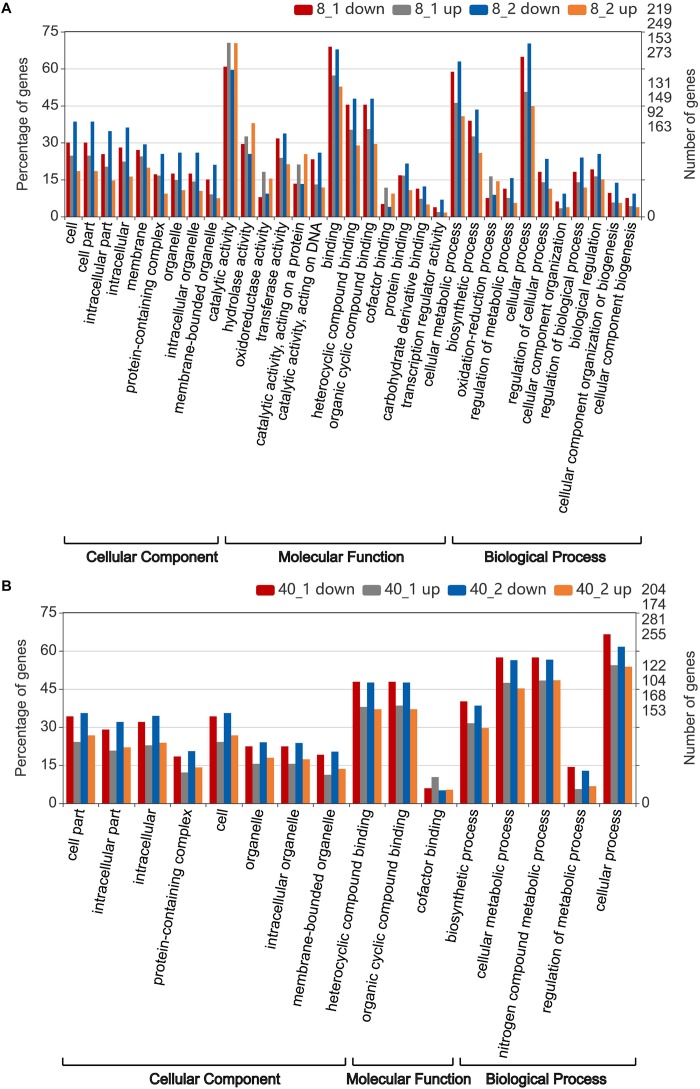
Gene ontology (GO) annotation of differentially expressed transcripts in the temperature stressed *O. furnacalis* transcriptome. Enriched GO analysis (*p* < 0.05) of differentially expressed transcripts were performed by pairwise comparison with the corresponding control group at 25°C. **(A)** The number of enriched GO term (*p* < 0.05) in the 8°C treated groups. **(B)** The number of enriched GO term (*p* < 0.05) in the 40°C treated groups. 8_1 up or down: Up or down-regulated transcripts 1 h posttreatment under 8°C; 8_2 up or down: Up or down-regulated transcripts 2 h posttreatment under 8°C; 40_1 up or down: Up or down-regulated transcripts 1 h posttreatment under 40°C; 40_2 up or down: Up- or down-regulated transcripts 2 h posttreatment under 40°C.

Further enrichment analysis showed that the transcripts enriched (*p*-value < 0.05) in “catalytic activity” term were significantly up-regulated after 2 h treatment in 8°C group, suggesting the *O. furnacalis* larvae were capable to accelerate energy metabolism under low temperature environment. While the transcripts enriched in “binding,” “cellular metabolic process” and “cell process” terms in the 8°C group were significantly down-regulated after 2 h treatment (Figure 3A). Similar to the transcripts enriched in the 8°C group, most transcripts enriched in the 40°C group were down-regulated (Figure 3B). However, the enriched GO terms in the 8°C group were more than that of the 40°C group (Figure 3), suggesting the response to the low temperature treatment was stronger than that to the high temperature treatment in *O. furnacalis* larvae, and many enriched terms related to cellular response terms under high temperature treatment were down-regulated, indicating the immune responses in high temperature treated groups were suppressed.

**FIGURE 4:**
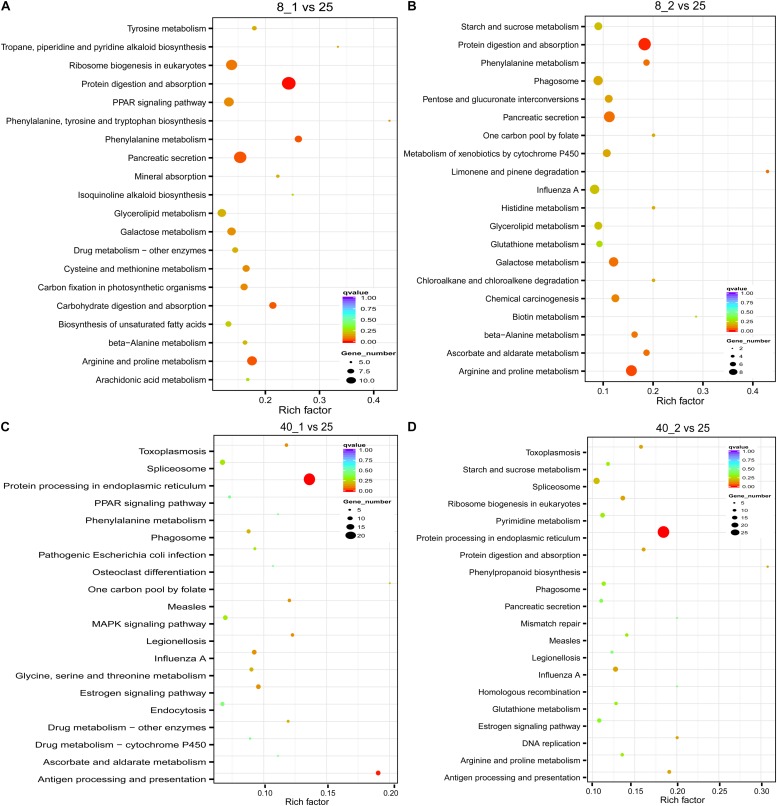
Distribution of KEGG functional groups within up- and down-regulated gene cohorts in the temperature stressed *O. furnacalis*. The bar chart corresponds to the matched entries of differentially expressed transcripts in their own functional category. 8_1 **(A)** and 8_2 **(B)**: The differentially expressed transcripts in the low temperature (8°C) treated group 1 and 2 h posttreatment, respectively; 40_1 **(C)** and 40_2 **(D)**: The differentially expressed transcripts in the high temperature (40°C) treated group 1 and 2 h posttreatment; 25_0: The differentially expressed transcripts in the normal temperature group under 25°C.

To discover the pathways of these differentially expressed transcripts involved in, the KEGG pathway analysis was performed (Xie et al., 2011). In the low temperature-treated group, protein digestion and absorption, and carbohydrate and amino acid related metabolisms such as “Galactose metabolism,” “Metabolism of xenobiotics by cytochrome P450,” “Arginine and proline metabolism” and “Phagosome” in which immune related protein lectin is involved, were enriched, suggesting the *O. furnacalis* larvae needed more energy consumption and stronger immune activity in responding to low temperature treatment (Figures 4A,B). However, the “Protein processing in endoplasmic reticulum,” “Spliceosome,” “MAPK signaling pathway,” “Estrogen signaling pathway,” and “Drug metabolism-cytochrome P450” were significantly enriched after treatment with high temperature. HSPs and lectins were also found to be involved in these pathways (Figures 4C,D).

**FIGURE 5:**
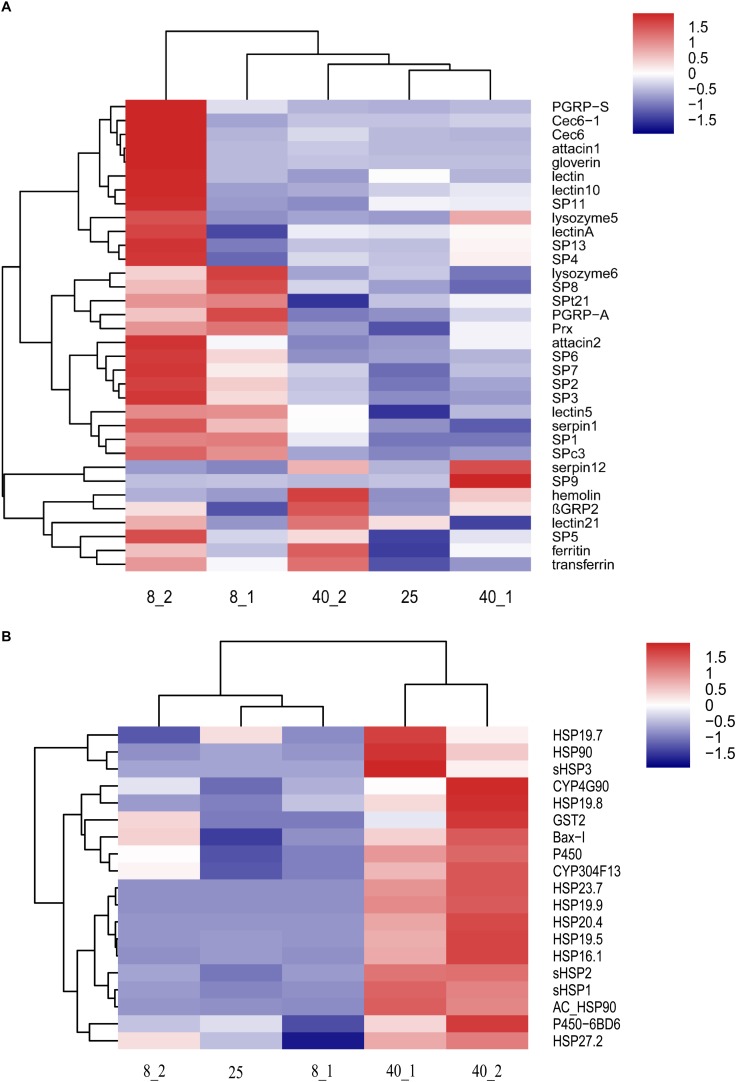
Hierarchical clustering analysis of immune- and stress-related genes in the temperatures stress treated *O. furnacalis* transcriptome. **(A)** Hierarchical clustering analysis of immunity-related genes in *O. furnacalis* transcriptome. **(B)** Hierarchical clustering analysis of stress response related genes in *O. furnacalis* transcriptome. 8_1 and 8_2: The differentially expressed transcripts in the low temperature (8°C) treated group 1 and 2 h posttreatment; 40_1 and 40_2: The differentially expressed transcripts in the high temperature (40°C) treated group 1 and 2 h posttreatment. 25_0: The differentially expressed transcripts in the normal temperature group under 25°C.

Based on the enrichment analysis, we found that the immune related transcripts such as peptidoglycan recognition proteins (*PGRP*s), serine proteases (*SP*s) and antibacterial peptides (*AMP*s), and stress response related transcripts such as heat shock proteins (*HSP*s), Glutathione-S-transferases (*GST*s) and *P450* were cold/hot-responsible genes under extreme temperature condition. These responsive genes were clustered by two-dimensional hierarchical clustering (Figures 5A,B). Compared with the expression patterns of immune related genes in high temperature treated group, most immune related transcripts in the low temperature treated groups were up-regulated, especially at 1 h posttreatment (Figure 5A). However, to defend against the high temperature treatment, *HSP*s and P450 related proteins were up-regulated in *O. furnacalis* larvae (Figure 5B). Interestingly, both *Ferritin* and *Transferrin* were up-regulated after 2 h treatment with low or high temperature (Figure 5A). Interestingly, we found *Bax inhibitor* was significantly up-regulated under 40°C stress, especially at 2 h posttreatment (Figure 5B). These data indicated that stronger immune responses might promote more resistance to the cold environment for *O. furnacalis* larvae while *HSP*s and P450 related proteins could protect *O. furnacalis* larvae from high temperature challenges.

### Expression Profile of Stress and Immune Response Related Genes in Response to Temperature Stress

**FIGURE 6:**
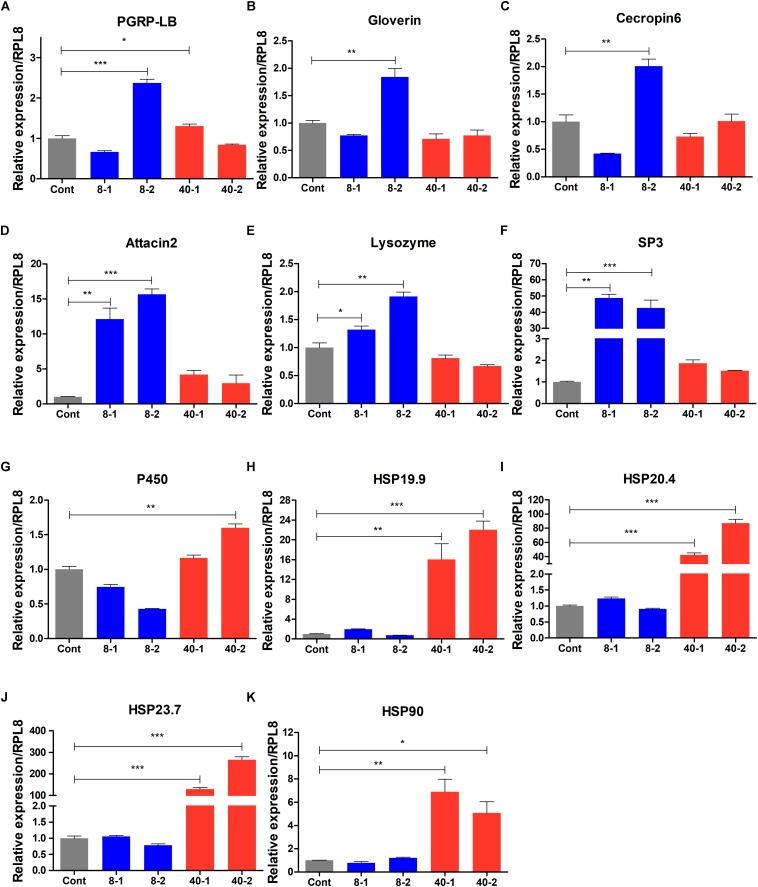
Expression profile of stress- and immune response related genes in the temperature stressed *O. furnacalis*. The relative expressions of *PGRP-LB* **(A)**, *Gloverin* **(B)**, *Cecropin*6 **(C)**, *Attacin*2 **(D)**, *Lysozyme* **(E)**, *SP*3 **(F)**, *P*450 **(G)**, *HSP*19.9 **(H)**, *HSP*20.4 **(I)**, *HSP*23.7 **(J)**, and *HSP*90 **(K)**. SP3: *serine protease* 3; Cont: control group reared under 25°C; 8_1 and 8_2: The differentially expressed transcripts in the low temperature (8°C) treated group 1 and 2 h posttreatment; 40_1 and 40_2: The differentially expressed transcripts in the high temperature (40°C) treated group 1 and 2 h posttreatment; Asterisks indicate significant differences: ^∗^*p* < 0.05; ^∗∗^*p* < 0.01; ^∗∗∗^*p* < 0.001 for pairwise comparisons by Student’s *t*-test.

To verify the FPKM value of RNA-seq data, we selected 5 stress response genes and 6 immune-related genes from heatmap (Figures 5A,B) to examine the expression patterns of these genes using qPCR, and found that the expression patterns of these genes were similar to the RNA-seq data (Figure 6), suggesting the RNA-seq data in this study are reliable. The immunity related genes such as *PGRP-LB*, *AMPs*, *Lysozyme*, and *Serine protease*-3 were significantly induced by low temperature (8°C), especially after 2 h treatment (Figures 6A–F). The up-regulation of these immunity related genes indicated that stronger immune responses were involved in the protection of *O. furnacalis* larvae from low temperature challenge. In response to high temperature challenge, *P450* and some genes of HSP family such as *HSP*19.9, *HSP*20.4, *HSP*23.7 and *HSP*90 were upregulated (Figures 6G–K), suggesting these genes may play as stress response genes to defend against the high temperature challenge.

### Temperature Stress Induced Changes in Hemolymph Iron Concentration

**FIGURE 7:**
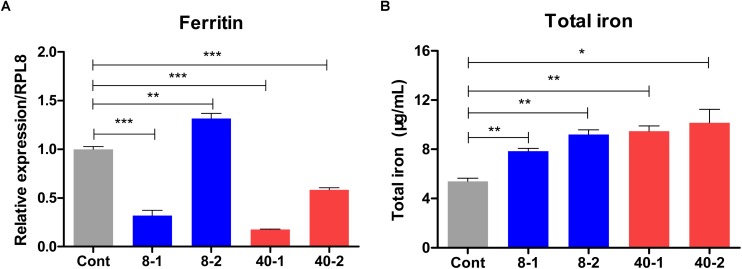
Expression profile of *Ferritin* gene and the change of total hemolymph iron concentration in the temperature stressed *O. furnacalis*. **(A)** The relative expression of *Ferritin*. **(B)** Total iron concentration changes in response to temperature stress. *O. furnacalis* larvae were reared under 8 or 40°C, and the hemolymph was collected for iron concentration determination. Cont: control group reared under 25°C; 8_1 and 8_2: The low temperature (8°C) treated group 1 and 2 h posttreatment; 40_1 and 40_2: The high temperature (40°C) treated group 1 and 2 h posttreatment. Asterisks indicate significant differences: ^∗^*p* < 0.05; ^∗∗^*p* < 0.01; ^∗∗∗^*p* < 0.001 for pairwise comparisons by Student’s *t*-test.

More interestingly, we found the iron storage protein *Ferritin* was also induced by low temperature stress after 2 h treatment, but inhibited in 40°C treated groups and 8°C treated group 1 h posttreatment (Figure 7A), we presumed that the expression of iron storage protein was inhibited so that more iron was released for host to defend against extreme temperature challenge. Based on the analyses of expression profiles, we found Ferritin may be involved in the responses to low and high temperature challenge. As an iron storage protein, the mRNA level changes of *Ferritin* could influence the iron concentration in hemolymph (Otho et al., 2016). To further confirm the qPCR results, the total iron concentration of hemolymph were determined according to the method described by Otho et al. (2016). The total iron concentrations in hemolymph were up-regulated after temperature stress, and changes of the total iron concentration in the hemolymph were in accord with the expression pattern of *Ferritin*, except the total iron concentration at 2 h posttreatment in 8°C treated groups (Figure 7B). The results were, in general, consistent with the RNA-seq and qPCR data, indicating that iron played important roles in defending against temperature stress.

## Discussion

During the life cycle of *O. furnacalis*, some larvae underwent extreme environmental changes in temperature during the hot summer and the cold winter. In the present study, we focused on the responses of *O. furnacalis* larvae to temperature stress, and uncovered the defense mechanism in responding to 8 and 40°C challenges.

In our study, we found that some immune related genes were up-regulated in the 8°C treated groups, indicating that up-regulated immune responses was helpful for *O. furnacalis* larvae to deal with cold stress. The similar investigation was found in larval *T. molitor* when the larvae were treated with 8°C, although the immune responses in cold stress groups were weaker than that in LPS treated groups (Catalan et al., 2012). However, most immune responses were triggered by pathogens after the pathogen associated molecular patterns (PAMPs) such as LPS and peptidoglycans from pathogens recognized by pattern recognition receptors (PRRs) of host immune system (Pal and Wu, 2009; Hoffmann and Akira, 2013; Chen and Lu, 2018; Wu et al., 2018). It’s still unclear that how the abiotic factor such as thermal stress presented to the immune system in insects. Therefore, there must be potential mechanism for insects to trigger the immune responses after receiving the signal of the cold and heat stress.

In this study, we also found some stress response related genes were up-regulated in the 40°C treated groups, and presumed that the up-regulated stress response related genes could contribute to the protection of *O. furnacalis* larvae from heat stress. These data suggested that the thermal damage from heat stress was weakened by stress response genes such as *P*450, *HSP*s and *GST*. Our results were similar to the findings in heat stressed *Aphidius gifuensis* (Kang et al., 2017). However, compared to other HSP families such as small *HSP*s, *HSP*60, and *HSP*90, *HSP*70 was not up-regulated in heat stressed *O. furnacalis* larvae in this study. To our knowledge, HSP70 included inducible HSP70 (heat shock 70) and constitutively expressed HSC70 (heat shock cognate 70) (Kiang and Tsokos, 1998). HSP70 family was the most conserved family among HSP families, and responded to various stresses (Bettencourt et al., 2008; Wang et al., 2019). Therefore, the unexpected results of HSP70 were invaluable to be further investigated. In addition, we found the expression patterns of *Bax inhibitor* were consistent with the expression patterns of *HSPs*. Our results were similar to the findings in wheat (Lu et al., 2018). Most reported studies on Bax inhibitor were focused on plants and mammals (Huckelhoven, 2004; Ishikawa et al., 2011), but the investigation on insects have not yet been reported. Based on our results, we inferred that Bax inhibitor in insects was involved in the responses to heat stress through the regulation of HSPs.

Reactive oxygen species (ROS) are indispensable for determining the fate of immune cells in both physiological and pathogenic environment, which were not only useful as effectors to defend against invading pathogens, but also harmful to cells and tissues in hosts (Nathan and Shiloh, 2000; Chen and Lu, 2018). ROS concentration in hosts could be up-regulated by both pathogenic infections and abiotic factors such as pesticides and thermal stress (Narendra et al., 2007; Yang et al., 2010; Pan et al., 2012). To balance the homeostasis of ROS, some antioxidant proteins such as GSTs, catalase, superoxide dismutase and peroxide dismutase were involved when ROS were excess (Corona and Robinson, 2006; Zhang and Lu, 2015). In the present study, we found the expression of *GST*2 was significantly enhanced under temperature stress, a result similar to that in *Panonychus citri* (Yang et al., 2010). We presume that GST2 plays important roles in antioxidant processes in thermal stressed *O. furnacalis* larvae.

Thermal stress on insects was always following the change of weather. Previously, few studies were focused on both antioxidant responses and immune responses in cold and heat stressed insects (Thomas and Blanford, 2003; Sinclair et al., 2013; King and MacRae, 2015; Kang et al., 2017; Wojda, 2017). In the present study, *O. furnacalis* larvae were shortly shocked with cold stress and heat stress, and we found the cold stress on *O. furnacalis* larvae could trigger stronger immune responses, while heat stress could up-regulate the expression of HSPs and P450 related proteins. The results indicate that immune responses and HSPs and P450 related proteins play important roles in defense against cold stress and heat stress, respectively.

Iron nutrition was required for the growth of insects and invading pathogens (Theil, 1987). In addition, as cofactors for other reactions, iron plays a crucial role in some physiological processes in both host and pathogens (Otho et al., 2016). Ferritin and transferrin were capable to regulate the concentration of iron in insects to kill the pathogens through limiting the iron uptake by invading pathogens (Geiser and Winzerling, 2012; Otho et al., 2016). In *Apis mellifera ligustica*, the expression levels of *Ferritin* could be regulated by high and low temperatures, and the expression levels of *Ferritin* in different tissues were regulated in different patterns (Ma et al., 2018). In our study, the expression of *Ferritin* was inhibited except in 8°C treated group 2 h posttreatment. Our results showed that the high or low temperature had strong impacts on the expression of *Ferritin* in *O. furnacalis* larvae, a result similar to that in *A. mellifera ligustica* (Ma et al., 2018). Further total iron assay shown that the total iron concentration was higher in 40°C treated groups and 8°C treated group 1 h posttreatment than that in the 8°C treated group 2 h posttreatment. These two results were almost consistent, suggesting that the regulation of iron concentration by Ferritin in thermal stressed *O. furnacalis* larvae might be an effective strategy for insects to survive. Based on our findings, we inferred that the release of iron from Ferritin could enhance the abilities of insects to respond to oxidative stress caused by changes in the internal and external environment of insects, and to increase the reaction activities of iron related proteins such as some antioxidant enzymes depending on iron, and then benefit the responses to temperature stress. The involvement of Ferritin in insect-stress and immunity will be further elucidated in following studies.

## Data Availability Statement

The RNA-Seq reads were deposited in the National Center for Biotechnology Information (NCBI) Sequence Read Archive (SRA) database. NCBI SRA records can be accessible with the following link: https://www.ncbi.nlm.nih.gov/sra/PRJNA551735.

## Author Contributions

CF designed the research. KC preformed the research. KC, TT, QS, ZW, KH, XL, JS, LW, and YY analyzed the data. CF and KC wrote the manuscript. All authors read and approved the final manuscript.

## Conflict of Interest

The authors declare that the research was conducted in the absence of any commercial or financial relationships that could be construed as a potential conflict of interest.

## Footnotes

1 https://www.ncbi.nlm.nih.gov/tools/primer-blast/
