# Supplementary material for: Transcription Analysis of the Stress and Immune Response Genes to Temperature Stress in Ostrinia furnacalis
Source: Front Physiol. 2019 Oct 15;10:1289. doi: 10.3389/fphys.2019.01289 (PMC6803539; doi:10.3389/fphys.2019.01289)
Supplement: Supplementary file 2 [file Data_Sheet_2.pdf]

Table S2. Length distribution of assembly

**Transcript length interval**	**200-500bp**	**500-1kbp**	**1k-2kbp**	**>2kbp**	**Total**
Number of transcripts	65364	20873	15818	15814	117869
Number of unigenes	52931	12775	8104	7657	81467

Table S3. Assembly statistics of reads

**Item**	**Min Length**	**Mean Length**	**Median Length**	**Max Length**	**N50**	**N90**	**Total Nucleotides**
Transcripts	201	968	430	29472	2004	332	114057975
Unigenes	201	786	352	29472	1636	276	64048359
